# Supplementary material for: An orphan protein of Fusarium graminearum modulates host immunity by mediating proteasomal degradation of TaSnRK1α
Source: Nat Commun. 2020 Sep 1;11:4382. doi: 10.1038/s41467-020-18240-y (PMC7462860; doi:10.1038/s41467-020-18240-y)
Supplement: Supplementary file 1 — Supplementary information [file 41467_2020_18240_MOESM1_ESM.pdf]

An orphan protein of *Fusarium graminearum* modulates host immunity by mediating proteasomal degradation of TaSnRK1α

Cong Jiang[1,2] , Ruonan Hei[1] ,Yang Yang[1], Shijie Zhang[3], Qinhu Wang[1], Wei Wang[1], Qiang Zhang[1], Min Yan[1], Gengrui Zhu[1], Panpan Huang[1], Huiquan Liu[1*] and Jin-Rong Xu[2*]

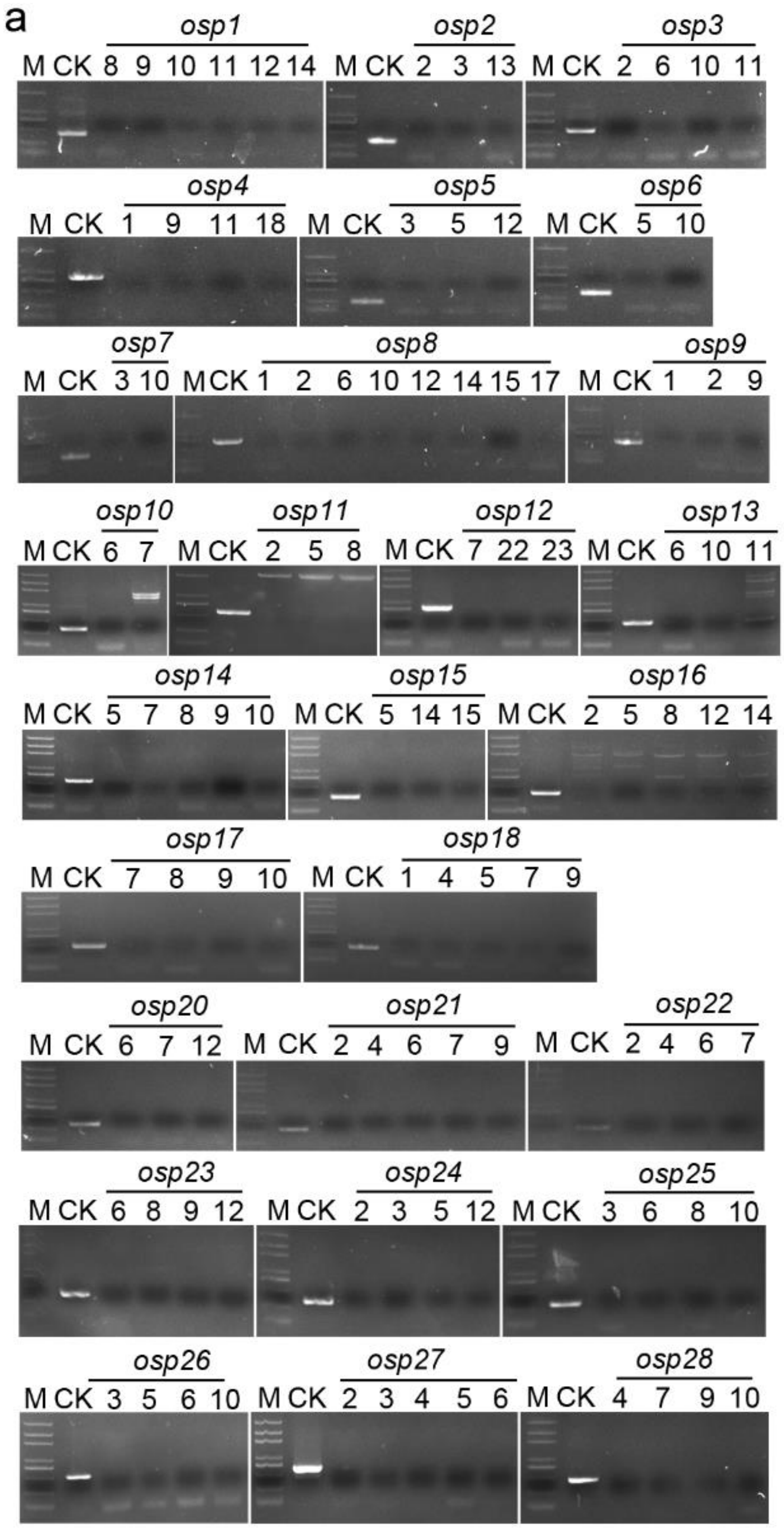
a
osp1
M CK 8 9 10 11 12 14
osp2
M CK 2 3 13
osp3
M CK 2 6 10 11
osp4
M CK 1 9 11 18
osp5
M CK 3 5 12
osp6
M CK 5 10
osp7
M CK 3 10
osp8
M CK 1 2 6 10 12 14 15 17
osp9
M CK 1 2 9
osp10
M CK 6 7
osp11
M CK 2 5 8
osp12
M CK 7 22 23
osp13
M CK 6 10 11
osp14
M CK 5 7 8 9 10
osp15
M CK 5 14 15
osp16
M CK 2 5 8 12 14
osp17
M CK 7 8 9 10
osp18
M CK 1 4 5 7 9
osp20
M CK 6 7 12
osp21
M CK 2 4 6 7 9
osp22
M CK 2 4 6 7
osp23
M CK 6 8 9 12
osp24
M CK 2 3 5 12
osp25
M CK 3 6 8 10
osp26
M CK 3 5 6 10
osp27
M CK 2 3 4 5 6
osp28
M CK 4 7 9 10

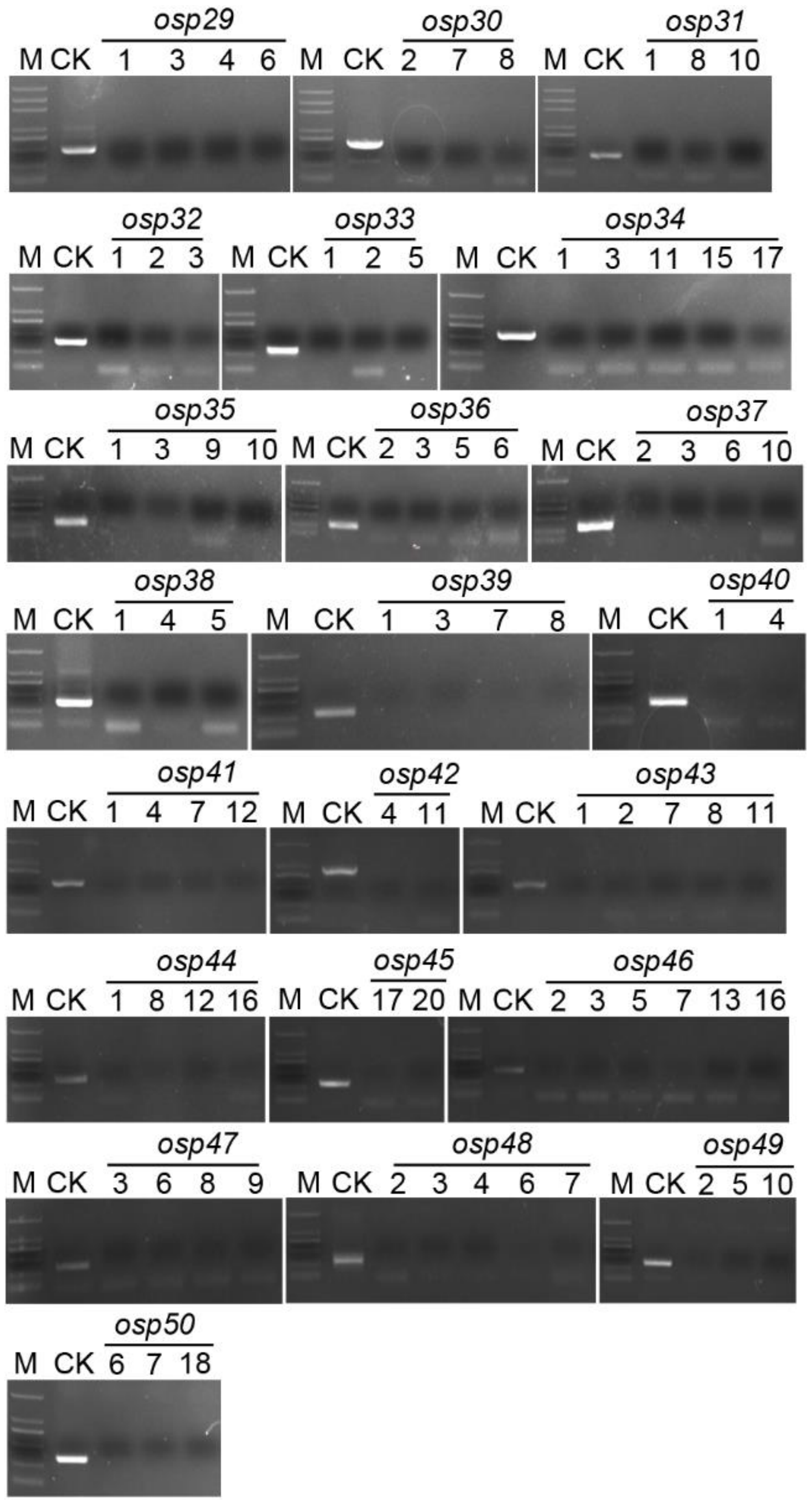

osp29
M CK 1 3 4 6
osp30
M CK 2 7 8
osp31
M CK 1 8 10
osp32
M CK 1 2 3
osp33
M CK 1 2 5
osp34
M CK 1 3 11 15 17
osp35
M CK 1 3 9 10
osp36
M CK 2 3 5 6
osp37
M CK 2 3 6 10
osp38
M CK 1 4 5
osp39
M CK 1 3 7 8
osp40
M CK 1 4
osp41
M CK 1 4 7 12
osp42
M CK 4 11
osp43
M CK 1 2 7 8 11
osp44
M CK 1 8 12 16
osp45
M CK 17 20
osp46
M CK 2 3 5 7 13 16
osp47
M CK 3 6 8 9
osp48
M CK 2 3 4 6 7
osp49
M CK 2 5 10
osp50
M CK 6 7 18

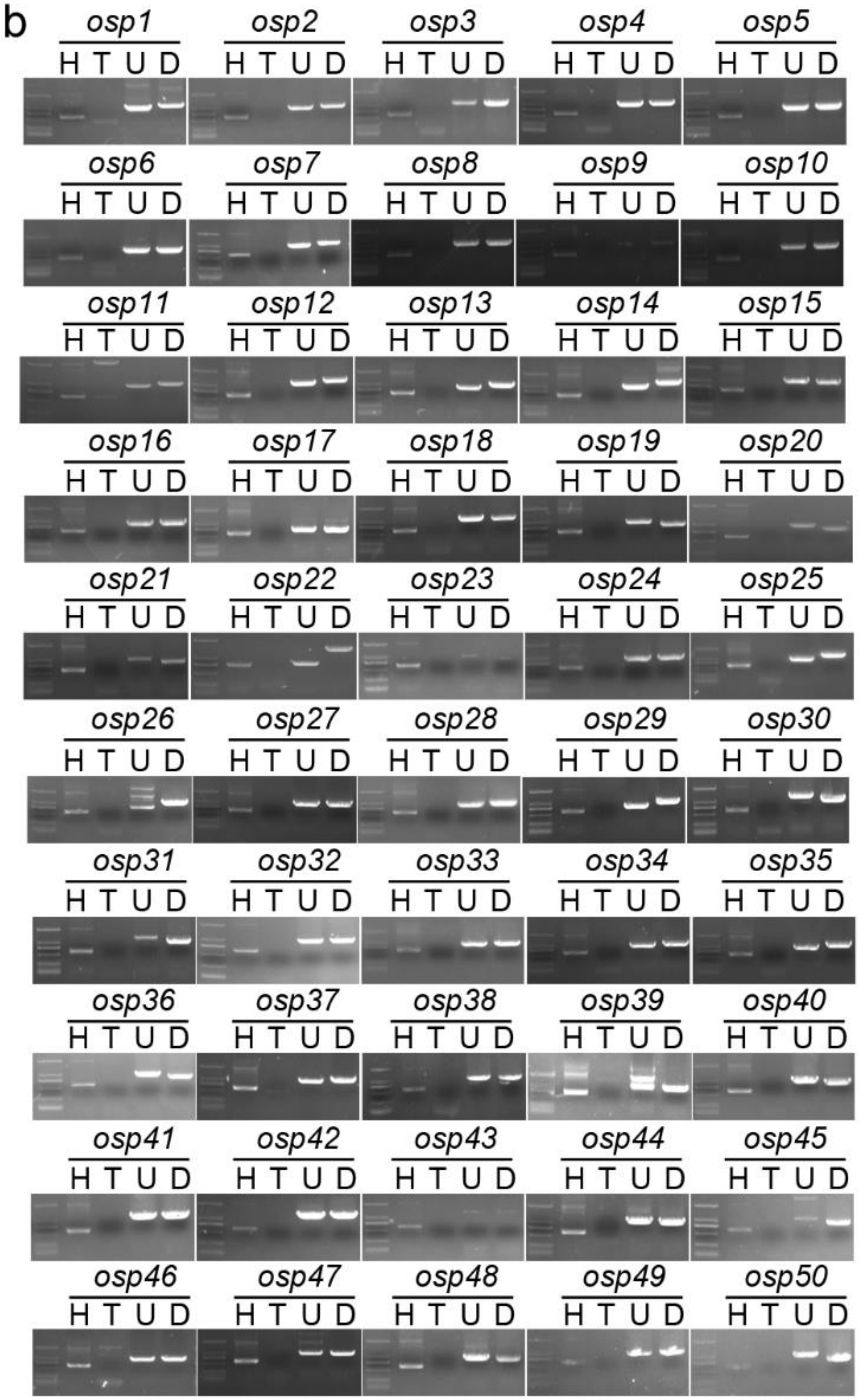
b
osp1
H T U D
osp2
H T U D
osp3
H T U D
osp4
H T U D
osp5
H T U D
osp6
H T U D
osp7
H T U D
osp8
H T U D
osp9
H T U D
osp10
H T U D
osp11
H T U D
osp12
H T U D
osp13
H T U D
osp14
H T U D
osp15
H T U D
osp16
H T U D
osp17
H T U D
osp18
H T U D
osp19
H T U D
osp20
H T U D
osp21
H T U D
osp22
H T U D
osp23
H T U D
osp24
H T U D
osp25
H T U D
osp26
H T U D
osp27
H T U D
osp28
H T U D
osp29
H T U D
osp30
H T U D
osp31
H T U D
osp32
H T U D
osp33
H T U D
osp34
H T U D
osp35
H T U D
osp36
H T U D
osp37
H T U D
osp38
H T U D
osp39
H T U D
osp40
H T U D
osp41
H T U D
osp42
H T U D
osp43
H T U D
osp44
H T U D
osp45
H T U D
osp46
H T U D
osp47
H T U D
osp48
H T U D
osp49
H T U D
osp50
H T U D

Supplementary Figure 1. Generation of gene replacement mutants of the *OSP* genes.

a. Deletion of the labelled *OSP* gene in the mutant strains listed in Supplementary Tables 1 was confirmed by the lack of PCR products amplified with primers 5F and 6R. M, molecular marker; CK, PH-1. **b.** PCR products amplified by primer pairs 7F/H4R (lane U) and H3F/8R (lane D) showed the occurrence of homologous recombination at the upstream and downstream flanking sequences of the labelled *OSP* gene, respectively, in the mutant strain selected for detailed analysis (marked in Supplementary Tables 1). Lane T was a repeat of PCR with primer pairs 5F and 6R to show the deletion of labelled *OSP* genes. Lane H showed the amplification of the *hph* gene with primers H1F and H2R. All the primers were described in Supplementary Table 7.

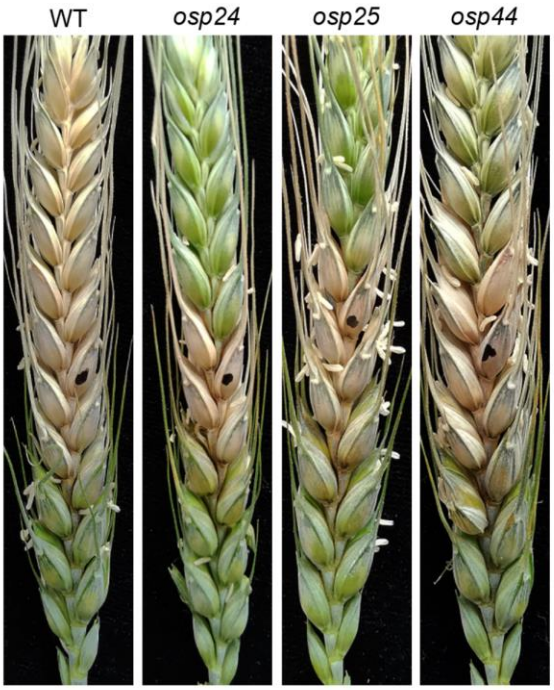

Supplementary Figure 2. Infection assays with wheat heads of cultivar Zhoumai 36.

Representative images of wheat heads of cultivar Zhoumai 36 infected with the wild type (PH-1) and the indicated *osp* mutants were photographed at 14 dpi.

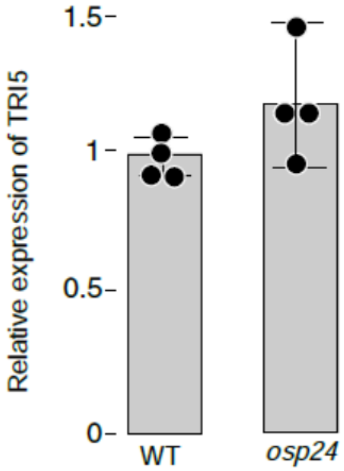

Supplementary Figure 3. Assays for *TRI5* expression by qRT-PCR.

Relative expression levels of *TRI5* were assayed by qRT-PCR with RNA isolated from inoculated and diseased spikelets from wheat heads inoculated with the wild type (WT) and *osp24* mutant. The expression level in WT-infected samples was arbitrarily set to 1. Mean and standard deviation were estimated with data from four (*n=4*) independent biological replicates (marked with black dots on the bars). No significant difference was observed based on Bootstrap analysis by BootstRatio (*P*=0.05).

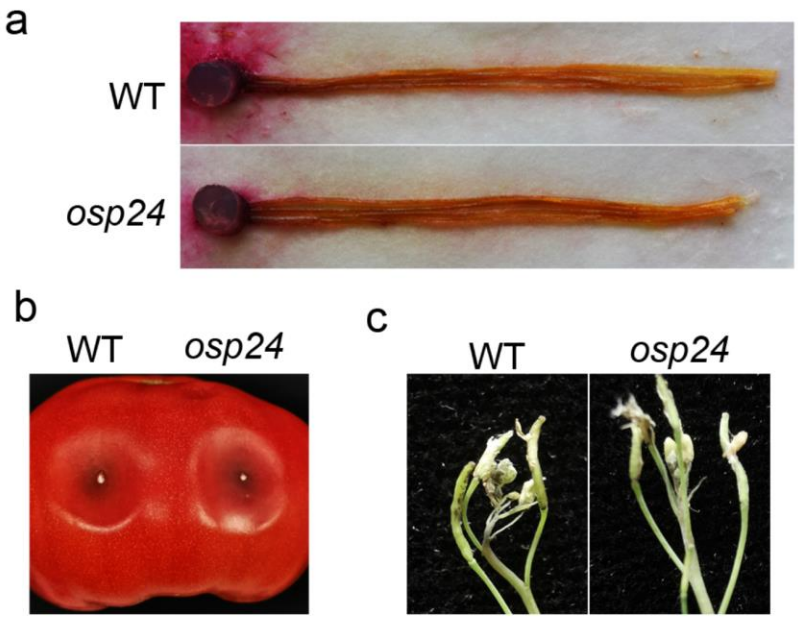

Supplementary Figure 4. Infection assays with corn silks, tomato fruits and Arabidopsis floral tissues.

Representative images of corn silks **(a),** tomato fruits **(b)** and Arabidopsis floral tissues **(c)** infected with the wild type (PH-1) and the *osp24* mutants were photographed at 7 dpi.

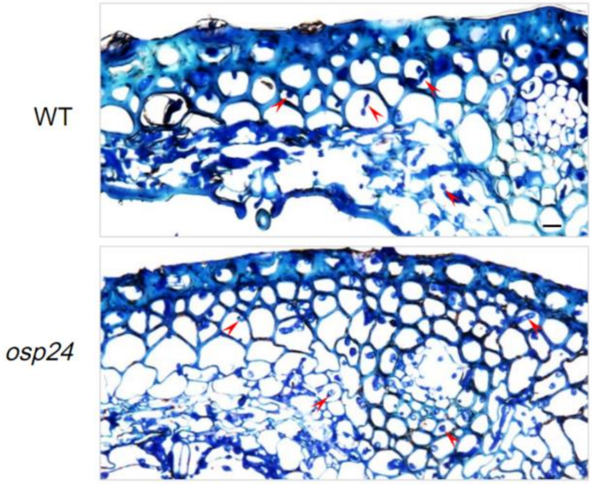

Supplementary Figure 5. Assays for invasive growth in wheat lemma

Wheat lemma were inoculated with the wild-type (WT) and *osp24* mutant strains and examined for invasive hyphae (marked with arrows) at 2 days post-inoculation (dpi). Scale bar, 20 μm.

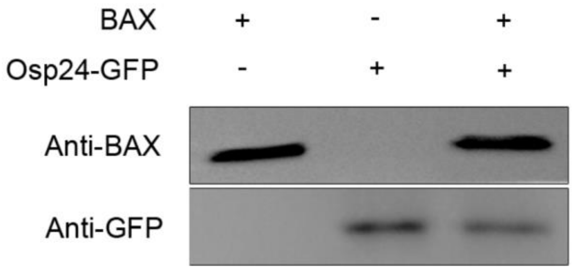

Supplementary Figure 6. Co-expression of BAX and Osp24-GFP in *N. benthamiana* leaves. Western blots of proteins isolated from *N. benthamiana* leaves expressing BAX and/or Osp24-GFP were detected with an anti-BAX or anti-GFP antibody.

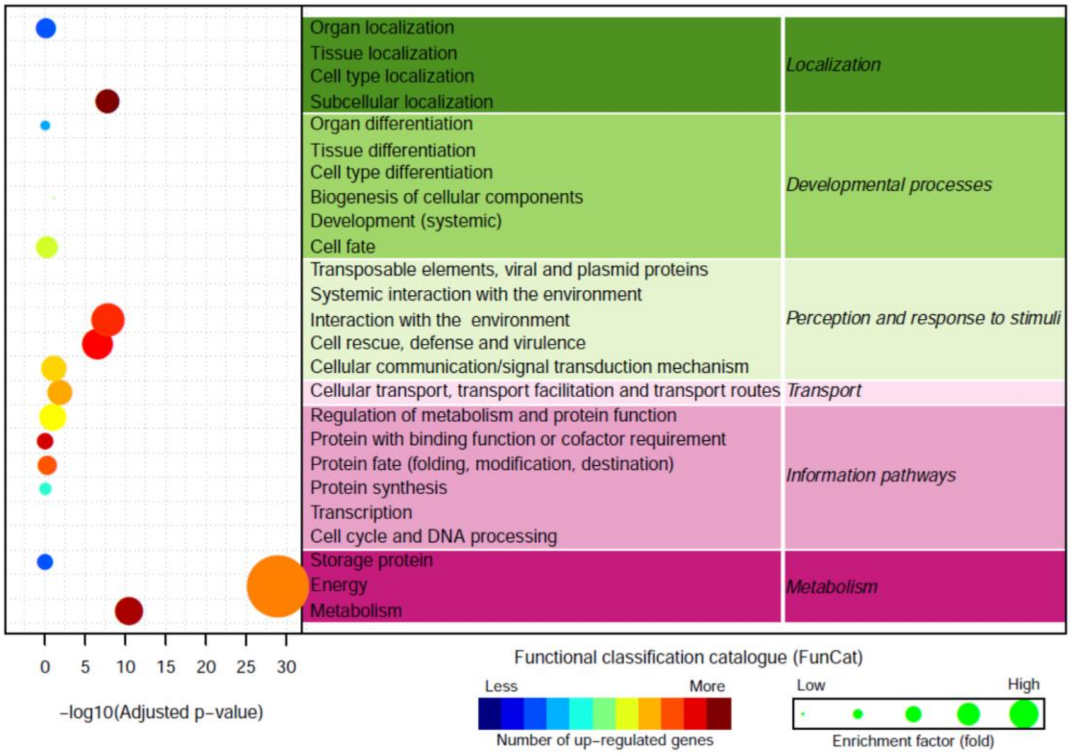

Supplementary Figure 7. FunCat enrichment analysis with DEGs up-regulated in *osp24*-infected wheat heads in comparison with wild type-inoculated samples. Genes functionally related to metabolism, information pathway, and perception and response to stimuli were significantly enriched.

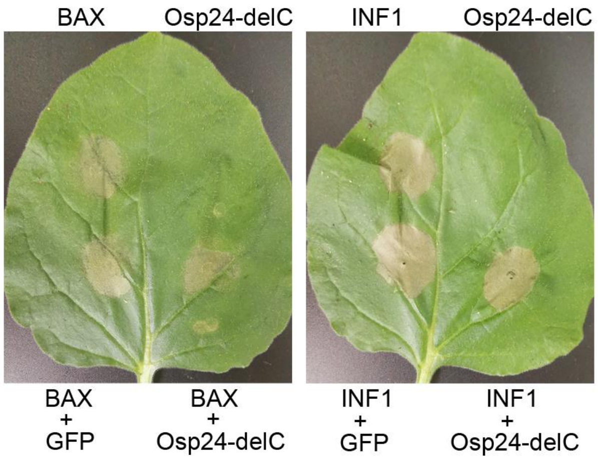

Supplementary Figure 8. Transient expression of truncated Osp24 failed to suppress programmed cell death triggered by BAX (left panel) or INF1 (right panel).

N. benthamiana leaves were infiltrated with Agrobacterium cells expressing GFP/BAX/INF1 alone or infiltrated with BAX/INF1-expressing cells at 18 h after infiltration with GFP or Osp24 truncated of its C-terminal region (Osp24-delC). Representative leaves were photographed at 5 days after infiltration.

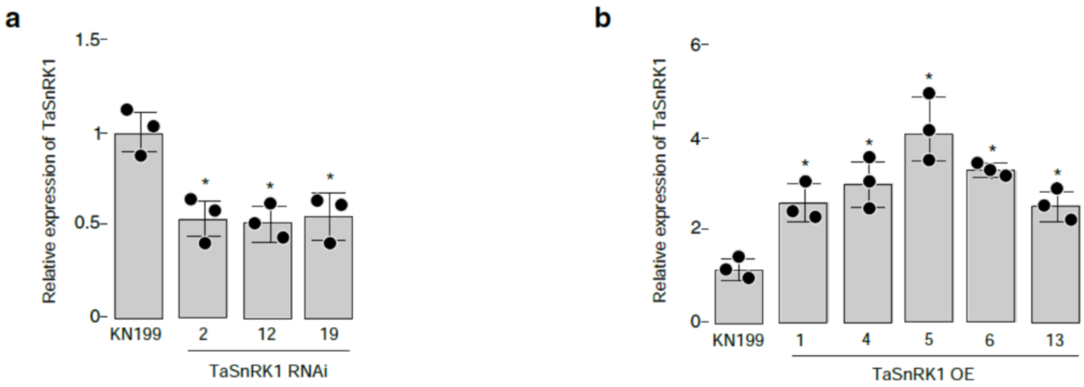

Supplementary Figure 9. Relative expression levels of TaSnRK1α in the three RNAi (**a**) and five overexpression (**b**) transgenic lines detected by qRT-PCR. Mean and standard deviation were estimated with data from three (*n=3*) independent replicates. Black dots on the bar represent the mean of each replicate. The asterisk *indicates significant differences (*P*=0.05) based on Bootstrap analysis by BootstRatio.

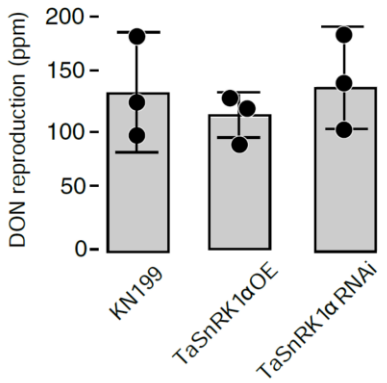

Supplementary Figure 10. DON levels in diseased wheat kernels of cultivar KN199 and its TaSnRK1α OE or TaSnRK1α RNAi transgenic plants.

DON was assayed with diseased wheat kernels inoculated with PH-1 that were collected from heads of cultivar KN199 and its TaSnRK1α OE or TaSnRK1α RNAi transgenic plants at 14 dpi. Mean and standard deviations of DON levels were calculated with data from three (*n=3*) independent replicates. No significant difference was observed based on ANOVA analysis followed by Duncan’s multiple range test (*P*=0.05).

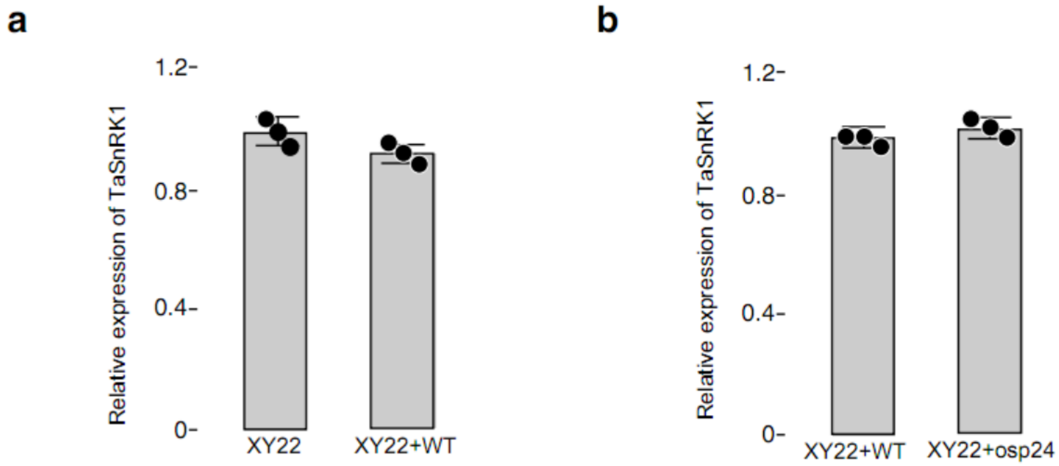

Supplementary Figure 11. Assays for the transcription levels of TaSnRK1α during *F. graminearum* infection by qRT-PCR.

a. Relative expression levels of TaSnRK1α in wheat heads of cultivar Xiaoyan 22 mock-inoculated with distilled water (XY22) or inoculated with PH-1 (XY22+WT). **b.** Relative expression levels of TaSnRK1α in wheat heads of cultivar Xiaoyan 22 mock-inoculated with distilled water (XY22) or inoculated with the *osp24* mutant (XY22+*osp24*). Mean and standard deviation were estimated with data from three (*n*=*3*) independent replicates. Black dots on the bar represent the mean of each replicate. No significant difference was observed based on Bootstrap analysis by BootstRatio (*P*=0.05).

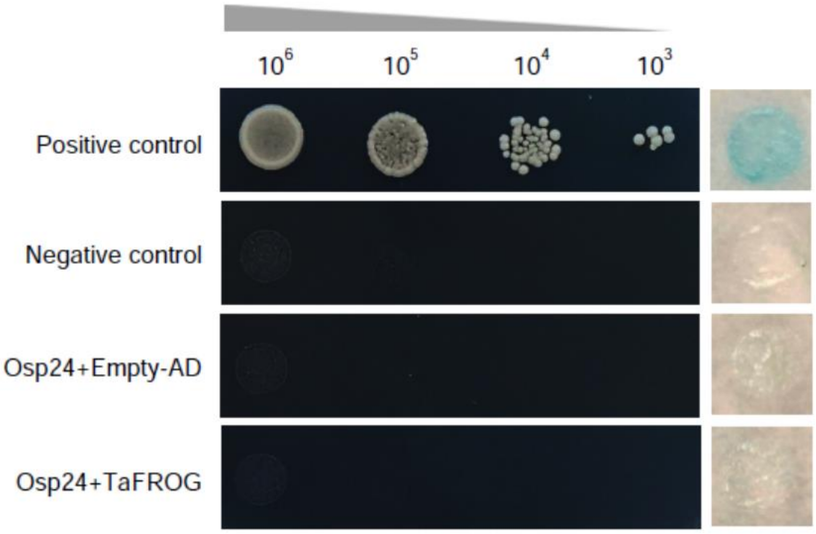

Supplementary Figure 12. Yeast two-hybrid assays for the interaction of Osp24 (Bait) with TaFROG (Prey). Different concentrations of the labeled yeast transformants were assayed for growth on SD-Trp-Leu-His plates and LacZ activity.

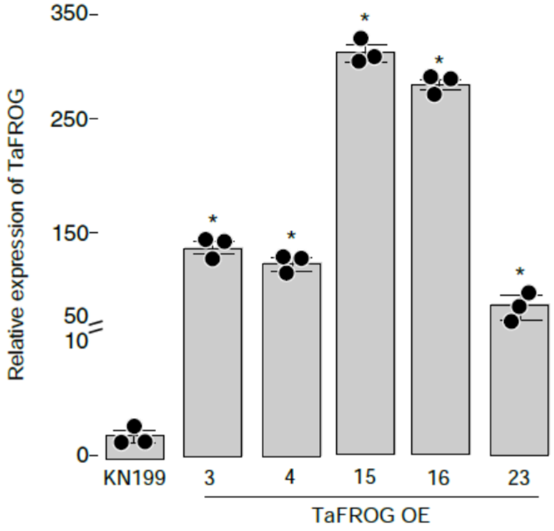

Supplementary Figure 13. Relative expression levels of TaFROG in five overexpression transgenic lines assayed by qRT-PCR. Mean and standard deviation were estimated with data from three (*n=3*) independent replicates. Black dots on the bar represent the mean of each replicate. The asterisk * indicates significant differences (*P*=0.05) based on Bootstrap analysis by BootstRatio analysis.

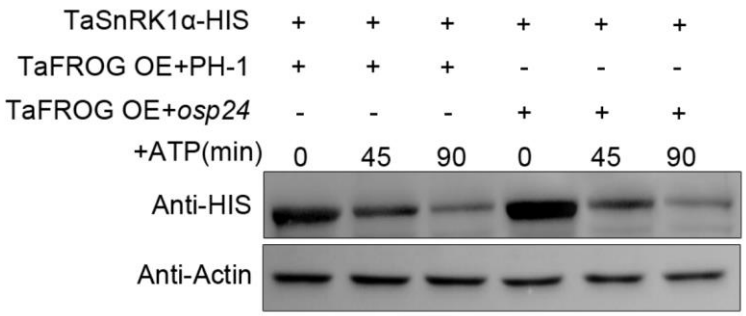

Supplementary Figure 14. Cell-free degradation assays with recombinant TaSnRK1α-HIS proteins.

Recombinant TaSnRK1α-HIS proteins were incubated with equal amounts of total proteins isolated from wheat heads of TaFROG OE transgenic plants (infected with PH-1 or the *osp24* mutant) for the indicated time after addition of 10 mM ATP. Western blots were detected with an anti-actin or anti-HIS antibody.

Supplementary Table 1. Deletion mutants of *OSP* genes generated in this study.

Gene name	Gene locus number	Number of mutant strains *
OSP1	FGSG_00770	8,**9**,10,11,12,14
OSP2	FGSG_01218	**2**,3,13
OSP3	FGSG_02021	**2**,6,10,11
OSP4	FGSG_02914	1,**9**,11,17,18
OSP5	FGSG_02957	**3**,5,12
OSP6	FGSG_03196	**5**,10
OSP7	FGSG_04687	**3**,10
OSP8	FGSG_04766	**1**,2,6,10,12,14,15,17
OSP9	FGSG_05241	**1**,2,5,9
OSP10	FGSG_06650	**6**,7
OSP11	FGSG_06780	2,**5**,8
OSP12	FGSG_07221	**7**,22,23
OSP13	FGSG_07403	**6**,7,10,11
OSP14	FGSG_07670	**5**,7,8,9,10
OSP15	FGSG_07972	**5**,7,14,15
OSP16	FGSG_08210	2,**5**,8,12,14
OSP17	FGSG_08332	7,**8**,9,10,12,16
OSP18	FGSG_08512	1,4,5,7,**9**
OSP19	FGSG_08908	4,**6**,9,11,12
OSP20	FGSG_10079	6,7,**12**
OSP21	FGSG_10112	**2**,4,6,7,9
OSP22	FGSG_11077	**5**,6,8
OSP23	FGSG_11418	**6**,8,9,12
OSP24	FGSG_11564	**2**,3,5,12
OSP25	FGSG_11647	**3**,6,8,10
OSP26	FGSG_11769	3,5,**6**,10
OSP27	FGSG_11910	**2**,3,4,5,6
OSP28	FGSG_12020	**4**,7,9,12
OSP29	FGSG_12045	**1**,3,4,6
OSP30	FGSG_12146	2,7,**8**
OSP31	FGSG_12249	1,8,**10**
OSP32	FGSG_12259	**1**,2,3
OSP33	FGSG_12722	1,2,**5**
OSP34	FGSG_12805	**1**,3,11,15,17
OSP35	FGSG_12822	1,3,**9**,10
OSP36	FGSG_12932	**2**,3,5,6
OSP37	FGSG_12981	2,3,**6**,10
OSP38	FGSG_13021	**1**,4,5
OSP39	FGSG_13067	1,3,7,**8**
OSP40	FGSG_13115	**16**,19
OSP41	FGSG_13244	1,4,**7**,12
OSP42	FGSG_13407	4,11
OSP43	FGSG_13412	1,2,7,**8**,11
OSP44	FGSG_13464	1,8,12,**16**
OSP45	FGSG_13505	17,20
OSP46	FGSG_13576	**2**,3,5,7,13,16
OSP47	FGSG_13635	**3**,6,8,9
OSP48	FGSG_13774	2,**3**,4,6,7
OSP49	FGSG_13782	**2**,5,10
OSP50	FGSG_13899	**6**,7,18

* The mutant strains selected for detailed analysis were in bold.

Supplementary Table 2. Phenotypes of the *osp* deletion mutants with reduced virulenc

Gene name	Disease index	Growth rate	Conidiation	Perithecium formation	DON production *
OSP4	78±11.8%	101.6±0.8%	92.4±1.7%	Normal	96.2±24.3%
OSP8	88.1±5.9%	98.4±0.8%	111±1.7%	Normal	103.7±13%
OSP13	87.3±9.7%	100.8±2.4%	98.3±12.7%	Normal	91.6±4.6%
OSP19	82.2±18.2%	99.2±2.4%	95.8±2.5%	Normal	90.2±9.4%
OSP24	49.1±4.7%	99.2±1.6%	104.2±13.6%	Normal	100.3±5%
OSP25	62.9±5.0%	99.2±3.2%	100±4.2%	Normal	97.5±5.6%
OSP29	84.7±22.8%	103.2±1.6%	96.7±7.6%	Normal	102.9±12.4%
OSP33	89.8±20.3%	97.6±2.4%	109.3±2.5%	Normal	93.9±2.8%
OSP36	86.4±16.9%	99.2±2.4%	90.7±3.4%	Normal	104.4±24.9%
OSP40	89±14.4%	101.6±0.8%	105.9±9.3%	Normal	99.7±13%
OSP44	64.4±7.4%	100±1.6%	95.8±11%	Normal	101.6±8.9%

*The disease index, growth rate, conidiation, perithecium formation, DON production were assayed with three independent replicates for all the strains. For each mutant, the percentage of changes was in comparison with PH-1 (arbitrarily set to 100%).

Supplementary Table 3. Expression profiles of the 50 *OSP* genes in *Fusarium graminearum*.

Gene name	Gene locus number	Hyphae_1*	Hyphae_2	Perithecia_1	Perithecia_2	Infection_1	Infection_2
OSP1	FGSG_00770	0.69	0.98	20.03	14.56	4.15	1.55
OSP2	FGSG_01218	0.00	0.00	0.00	0.00	0.00	0.00
OSP3	FGSG_02021	0.68	0.58	2.85	3.51	2.20	2.27
OSP4	FGSG_02914	0.08	0.32	0.00	0.00	47.74	52.12
OSP5	FGSG_02957	0.39	0.00	0.00	0.00	0.00	0.00
OSP6	FGSG_03196	0.26	0.00	0.73	0.84	0.94	0.00
OSP7	FGSG_04687	0.00	0.00	0.00	0.23	0.00	0.00
OSP8	FGSG_04766	0.00	0.00	8.28	6.63	0.00	0.00
OSP9	FGSG_05241	0.12	0.06	0.81	0.61	0.10	0.05
OSP10	FGSG_06650	0.36	0.77	0.51	1.17	0.97	0.30
OSP11	FGSG_06780	0.00	0.27	0.00	0.21	0.00	0.00
OSP12	FGSG_07221	2.81	1.51	2.26	4.89	7.99	8.78
OSP13	FGSG_07403	0.00	0.00	0.00	0.00	0.00	0.00
OSP14	FGSG_07670	1.18	1.16	2.07	1.91	5.85	5.94
OSP15	FGSG_07972	0.00	0.22	0.00	0.34	0.37	0.00
OSP16	FGSG_08210	0.00	0.18	0.00	0.00	31.33	25.01
OSP17	FGSG_08332	0.00	0.00	0.25	0.19	0.00	0.00
OSP18	FGSG_08512	0.39	0.42	1.93	2.23	1.77	3.30
OSP19	FGSG_08908	0.00	0.38	0.40	0.11	0.64	0.95
OSP20	FGSG_10079	0.00	0.00	0.45	0.52	0.29	0.00
OSP21	FGSG_10112	1.25	1.78	3.79	5.73	0.75	1.05
OSP22	FGSG_11077	1.02	0.14	0.54	0.62	8.30	7.72
OSP23	FGSG_11418	1.42	2.19	0.22	0.00	0.00	0.00
OSP24	FGSG_11564	157.46	17.35	0.26	0.30	290.77	310.82
OSP25	FGSG_11647	0.00	0.00	0.00	0.35	39.41	54.24
OSP26	FGSG_11769	0.00	0.13	0.33	0.39	0.00	0.80
OSP27	FGSG_11910	6.85	3.30	0.48	1.11	0.31	0.00
OSP28	FGSG_12020	0.65	0.47	5.67	4.25	1.97	1.10
OSP29	FGSG_12045	0.00	0.25	0.00	0.38	0.00	0.00
OSP30	FGSG_12146	0.38	0.21	4.31	1.25	1.04	2.58
OSP31	FGSG_12249	3.36	2.64	1.89	0.36	0.40	0.00
OSP32	FGSG_12259	0.00	0.00	0.00	0.77	0.00	0.40
OSP33	FGSG_12722	2.66	2.09	3.49	2.30	0.96	0.89
OSP34	FGSG_12805	0.00	0.00	0.00	0.00	0.00	0.00
OSP35	FGSG_12822	0.00	0.00	0.00	0.00	0.00	0.29
OSP36	FGSG_12932	9.50	10.17	10.84	17.35	2.14	3.99
OSP37	FGSG_12981	1.52	4.06	23.43	23.80	2.74	10.19
OSP38	FGSG_13021	0.00	0.00	1.52	2.11	0.00	0.00
OSP39	FGSG_13067	0.25	0.27	2.13	0.41	0.00	0.00
OSP40	FGSG_13115	0.45	0.00	1.89	4.73	2.02	0.75
OSP41	FGSG_13244	0.00	0.00	0.88	0.00	0.00	0.00
OSP42	FGSG_13407	0.10	0.07	29.96	26.24	2.78	3.34
OSP43	FGSG_13412	1034.63	1279.47	4.63	2.56	2.07	0.72
OSP44	FGSG_13464	1.12	4.20	26.01	35.06	1109.59	1087.15
OSP45	FGSG_13505	0.00	0.00	7.43	2.24	71.86	53.31
OSP46	FGSG_13576	1.09	0.50	0.66	0.00	1.12	1.31
OSP47	FGSG_13635	0.23	0.00	0.00	0.37	0.42	0.00
OSP48	FGSG_13774	1.37	0.24	0.96	0.37	1.23	0.00
OSP49	FGSG_13782	0.26	0.00	1.83	3.81	49.40	48.13
OSP50	FGSG_13899	0.00	0.00	0.00	0.35	0.00	0.00

* TPM (Transcripts Per Kilobase Million) values from RNA-seq data of two replicates of vegetative hyphae (Hyphae), 3-dpf perithecia (perithecia), and infected wheat heads (Infection) from two previous studies (Liu et al., 2016; Jiang et al., 2019).

Supplementary Table 4. Osp24-interacting clones (OIC) identified by yeast two-hybrid library screening.

Name	Clonies	Annotation
TaSnRK1 (OIC1)	4,6,11,18,23	SNF1-related protein kinase 1
OIC2	2,15,19,20	SGT1 (suppressor of the G2 allele of *skp1*
OIC3	1	S-acyltransferase 23
OIC4	8	Peroxisome biogensis protein 5
OIC5	14	Ribosomal protein L7
OIC6	5,30	Phosphoglycolate phosphatase
OIC7	25	Blue copper-binding protein
OIC8	12	Pre-mRNA-splicing factor SLU7
