# Supplementary material for: An orphan protein of Fusarium graminearum modulates host immunity by mediating proteasomal degradation of TaSnRK1α
Source: Nat Commun. 2020 Sep 1;11:4382. doi: 10.1038/s41467-020-18240-y (PMC7462860; doi:10.1038/s41467-020-18240-y)
Supplement: Supplementary file 3 — Description of Additional Supplementary Files [file 41467_2020_18240_MOESM3_ESM.docx]

**Description of Additional Supplementary Files**

File Name: Supplementary Data 1

Description: GO enrichment analysis with DEGs up-regulated in *osp24*-infected wheat heads in comparison with wild type-inoculated samples.

File Name: Supplementary Data 2

Description: Identification of fungal DEGs in *osp24*-infected wheat heads in comparison with wild type-inoculated samples.

File Name: Supplementary Data 3

Description: Primers used to generate *osp* deletion mutants.
