# Supplementary material for: An orphan protein of Fusarium graminearum modulates host immunity by mediating proteasomal degradation of TaSnRK1α
Source: Nat Commun. 2020 Sep 1;11:4382. doi: 10.1038/s41467-020-18240-y (PMC7462860; doi:10.1038/s41467-020-18240-y)
Supplement: Supplementary file 7 — Reporting Summary [file 41467_2020_18240_MOESM7_ESM.pdf]

nature research

Corresponding author(s): Jin-Rong Xu

Reporting Summary

Nature Research wishes to improve the reproducibility of the work that we publish. This form provides structure for consistency and transparency in reporting. For further information on Nature Research policies, see Authors & Referees and the Editorial Policy Checklist.

Statistical parameters

When statistical analyses are reported, confirm that the following items are present in the relevant location (e.g. figure legend, table legend, main text, or Methods section).

n/a	Confirmed	
	x	The exact sample size (*n*) for each experimental group/condition, given as a discrete number and unit of measurement
	x	An indication of whether measurements were taken from distinct samples or whether the same sample was measured repeatedly
	x	The statistical test(s) used AND whether they are one- or two-sided<br>*Only common tests should be described solely by name; describe more complex techniques in the Methods section.*
x		A description of all covariates tested
x		A description of any assumptions or corrections, such as tests of normality and adjustment for multiple comparisons
	x	A full description of the statistics including central tendency (e.g. means) or other basic estimates (e.g. regression coefficient) AND variation (e.g. standard deviation) or associated estimates of uncertainty (e.g. confidence intervals)
	x	For null hypothesis testing, the test statistic (e.g. *F*, *t*, *r*) with confidence intervals, effect sizes, degrees of freedom and *P* value noted<br>*Give P values as exact values whenever suitable.*
x		For Bayesian analysis, information on the choice of priors and Markov chain Monte Carlo settings
x		For hierarchical and complex designs, identification of the appropriate level for tests and full reporting of outcomes
x		Estimates of effect sizes (e.g. Cohen's *d*, Pearson's *r*), indicating how they were calculated
	x	Clearly defined error bars<br>*State explicitly what error bars represent (e.g. SD, SE, CI)*

Our web collection on statistics for biologists may be useful.

Software and code

Policy information about availability of computer code

Data collection	Bio-Rad CFX manager for qPCR data collection and analysis.
Data analysis	The RNA-seq reads of PH-1 and osp24 infected wheat head were mapped to reference genomes via hisat2, and the expression level of each gene was count by using featureCounts. Differentially expressed genes (DEGs) were identified by edgeRun. Functional Catalogue (FunCat) and Gene Ontology (GO) enrichment annotations were performed with custom scripts and Blast2GO, respectively.

For manuscripts utilizing custom algorithms or software that are central to the research but not yet described in published literature, software must be made available to editors/reviewers upon request. We strongly encourage code deposition in a community repository (e.g. GitHub). See the Nature Research guidelines for submitting code & software for further information.

Data

Policy information about availability of data

All manuscripts must include a data availability statement. This statement should provide the following information, where applicable:

- Accession codes, unique identifiers, or web links for publicly available datasets
- A list of figures that have associated raw data
- A description of any restrictions on data availability

All data generated or analysed during this study are included in this published article (and its supplementary information files).

April 2018

Field-specific reporting

Please select the best fit for your research. If you are not sure, read the appropriate sections before making your selection.

- [x] Life sciences
- [ ] Behavioural & social sciences
- [ ] Ecological, evolutionary & environmental sciences

For a reference copy of the document with all sections, see nature.com/authors/policies/ReportingSummary-flat.pdf

Life sciences study design

All studies must disclose on these points even when the disclosure is negative.

Sample size	When assays for disease index, mean and standard deviation of the disease index of each strain were estimated with data from three (n=3) independent experiments with at least 10 biological replicates (n≥10 infected wheat heads) examined for each experiment. When assays for infection cushions formation and infectious growth, mean and standard deviation were estimated with data from three independent biological replicates (n=3). For infiltration assays with N. benthamiana leaves, each infiltration experiment was repeated at least three times (n=3) with a minimum of three leaves (n≥3) tested. Results from three independent biological replicates (n=3) were used to estimate the ratio of wheat and F. graminearum biomasses. For cell-free protein degradation assays, each experiment was repeated at least three times (n≥3).
Data exclusions	No data were excluded from the analyses
Replication	All attempts at replication were successful. The number of replicates is indicated in the corresponding figure legend and/or in the corresponding material and method section.
Randomization	Randomization was used for all the biological experiments.
Blinding	No blinding was done as none of the experiments described in this study involve group allocation during data collection or analyses.

Reporting for specific materials, systems and methods

Materials & experimental systems

n/a	Involved in the study	
[ ]	[x]	Unique biological materials
[ ]	[x]	Antibodies
[x]	[ ]	Eukaryotic cell lines
[x]	[ ]	Palaeontology
[x]	[ ]	Animals and other organisms
[x]	[ ]	Human research participants

Methods

n/a	Involved in the study	
[x]	[ ]	ChIP-seq
[x]	[ ]	Flow cytometry
[x]	[ ]	MRI-based neuroimaging

Unique biological materials

Policy information about availability of materials

Obtaining unique materials	All unique materials used are readily available from the corresponding authors.

Antibodies

Antibodies used	anti-HIS and anti-GST antibodies (CoWin Bioscience Co., China); anti-RBX1 and anti-20s antibodies (Abcam, UK); anti-actin antibodies (Easybio, China); anti-BAX antibody (Beyotime Biotechnology; China)
Validation	Data provided in the manuscript confirmed specific reactivity of these antibodies in Fusarium graminearum

April 2018
